# Improving the Structural Efficiency of Punched-Metal-Material-Based Composites

**DOI:** 10.3390/polym16243468

**Published:** 2024-12-12

**Authors:** Mihails Lisicins, Dmitrijs Serdjuks, Pavel Akishin, Viktors Mironovs, Vadims Goremikins, Vjaceslavs Lapkovskis

**Affiliations:** 1 Faculty of Civil and Mechanical Engineering, Institute of Civil Engineering, Riga Technical University, LV-1011 Riga, Latvia; mihails.lisicins@gmail.com (M.L.); dmitrijs.serdjuks@rtu.lv (D.S.); vadims.goremikins@rtu.lv (V.G.); 2 Faculty of Civil and Mechanical Engineering, Institute of High-Performance Materials and Structures, Riga Technical University, LV-1011 Riga, Latvia; pavels.akisins@rtu.lv; 3 Faculty of Civil and Mechanical Engineering, Mechanical and Biomedical Engineering Institute, Riga Technical University, LV-1011 Riga, Latvia; viktors.mironovs@rtu.lv; 4 Faculty of Civil and Mechanical Engineering, Aeronautics, Space Engineering and Transport Institute, Riga Technical University, LV-1011 Riga, Latvia

**Keywords:** punched-metal materials, recycling, industrial wastes, tensioned load-carrying structural members, FEM analysis, coefficient of stress concentration

## Abstract

This study investigates the potential of reusing punched-steel waste, a significant component of solid inorganic waste, in composite materials for construction applications. Driven by the growing global demand for raw materials (which is projected to quadruple by 2050) and the need for sustainable waste management practices, this research explores the creation of a composite material (PPLK) incorporating punched-steel tape (LPM-4 grade) embedded in a polypropylene matrix. Experimental testing of PPLK specimens (310 × 90 × 6.30 mm) and finite element analysis (FEA) were employed to evaluate the mechanical properties and stress concentration coefficient. The results show that the PPLK composite exhibits a load-carrying capacity of 21.64 kN, exceeding the sum of its individual components by 11.37%, demonstrating a synergistic effect between the steel (average tensile strength 220.65 MPa) and polypropylene. FEA further revealed that increasing the matrix’s modulus of elasticity to 42 MPa significantly reduces the stress concentration coefficient in the steel component, resulting in a 24% enhancement of the elastic force. The findings suggest a viable path toward sustainable waste management and improved material utilisation in the construction industry.

## 1. Introduction

**Figure 1:**
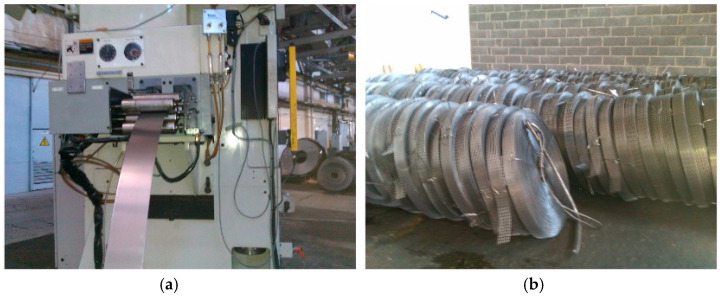
Punched-steel waste from the stamping process in mechanical engineering: (**a**)—Steel tape orientation in the stamping machine; (**b**)—Punched-steel waste in coil form after stamping of drive chain components.

The sustainable use of natural resources and waste recycling from economic activities are two primary environmental goals. The directive set forth in [1] establishes the fundamental concepts and definitions underpinning waste management practices throughout the European Union. It emphasises the principles of recycling and recovery, laying the foundation for effective waste processing in member states. The regulation set forth in [2] outlines the criteria for determining when specific scrap metals are no longer classified as waste. This is crucial for the facilitation of recycling operations and clarifying the legal status of recycled materials under the Waste Framework Directive. Studies show that global raw-material consumption reached 90 billion tonnes in 2017 and may quadruple by 2050 [3]. At the same time, a significant portion of solid inorganic waste is composed of various metallic materials. Much waste from punched steel is in tape form rather than sheets. In mechanical engineering, this material is generated during the stamping of components (Figure 1).

Technologies for the recycling and repurposing of metallic materials are advancing; however, studies conducted by the European Commission indicate that the rise in material recovery is negligible relative to the escalating demand for metallic resources. Consequently, there is a pressing necessity for heightened public awareness and the innovation of new solutions for the reutilization of metallic materials.

The building industry is expanding swiftly due to the rising demand for materials. The construction sector has been identified as a priority area for this transition, not only because it is fundamental to the economic system, providing approximately 9% of the EU’s GDP, but also because it is a significant source of employment [4]. The construction sector can thus catalyse the more efficient utilisation of raw materials and the resolution of recycling issues relating to metal and other waste. The primary strategies for enhancing the efficiency of metal raw-material utilisation include advancing recycling technologies, optimising manufacturing structures during the design phase, and re-utilising scrap, of which up to 90% can be reintegrated into the life cycle.

The Circular Economy (CE) and the 3Rs strategy (Reduce, Reuse, and Recycle) have gained significant traction recently, particularly in the manufacturing and construction industries. This approach addresses the growing global waste generation problem, with waste projected to increase from 2.02 billion tons in 2016 to 3.4 billion tons by 2050 [5]. In the steel sector, there is a growing emphasis on reusing and recycling byproducts to achieve the ambitious “zero waste” goal [6]. This trend is particularly relevant given the large quantities of metal scraps, including iron and steel, generated from manufacturing-based machining processes [7]. Innovative approaches to steel waste recycling are also being explored. For instance, the use of punched-metal straps as replacements for main steel rebars in regular concrete beams has been investigated, demonstrating the viability and advantages of this alternative [8]. Furthermore, research is being conducted on the sustainable lifecycle management of perforated metal materials, and this has resulted in the proposal of new technologies for recycling or reuse [9]. This reflects a broader trend towards finding creative and sustainable applications for various forms of steel waste. Several research groups are investigating composite materials comprising a reinforcing structure composed of fibres embedded in different polymers as a matrix. Fiber-reinforced polymers (FRPs) have emerged as a promising alternative to traditional steel reinforcement in various construction applications. FRP bars as compression reinforcement in columns have gained significant attention. One critical review [10] highlights the potential of FRP-reinforced columns and suggests that code authorities should recognise the use of FRP in compression members. This recommendation is based on an analysis of existing research and the accuracy of various design approaches in predicting FRP-reinforced column behaviour. FRP composites have demonstrated versatility in strengthening, repairing, and retrofitting multiple structures. In [11], a review provides an overview of FRP composites used as strengthening materials in the construction industry, focusing on their engineering aspects and design characteristics. The review concludes that FRP techniques will likely remain preferred for numerous projects involving bridges, buildings, and historical monuments. The application of FRP composites extends to both new and existing concrete structures. The work in [12] discusses the use of FRP composites as reinforcement in concrete structures, noting their effectiveness in increasing the flexural capacity of reinforced concrete column–beam joints. Experimental studies have further validated the benefits of FRP in structural applications. The study related in [13] investigated the behaviour of FRP-steel-confined concrete columns under reversed cyclic lateral loads. The research demonstrated that increasing the number of FRP layers improved various structural behaviours, including the yield load and displacement, peak load and displacement, ultimate load and displacement, and ductility coefficient. The applications of FRP composites extend beyond reinforcement and strengthening. The study in [14] focuses on the structural applications of FRP composites as major load-carrying members in aggressive environments, demonstrating their versatility and durability in challenging conditions.

While FRP offers numerous advantages, steel remains widely used in industrial applications due to its excellent mechanical properties. However, steel’s poor corrosion resistance in chloride-containing environments is a significant drawback. The work in [15] explores the use of epoxy-based polymer coatings as an effective anti-corrosive solution for steel, highlighting their unique mechanical properties, superior adhesion, good thermal stability, and excellent corrosion and chemical resistance. The use of fibre-reinforced polymer (FRP) composites and steel–polymer sandwich structures in civil engineering applications has gained significant attention due to the unique properties and potential advantages of this approach, compared to traditional materials. This review synthesises recent research on these advanced materials, focusing on their structural applications, corrosion resistance, and mechanical properties. FRP composites have shown promise in various structural applications, particularly column members. The work in [16] provides a comprehensive review of FRP materials used in column applications for new and existing construction, identifying knowledge gaps and areas for further research. This work highlights the growing interest in FRP as an alternative to traditional construction materials. One of the key advantages of FRP composites is their corrosion resistance. The work in [17] investigates the structural behaviour and corrosion resistance of hybrid FRP-wrapped steel bars for reinforced concrete structures. The study demonstrates that FRP-wrapped steel reinforcing bars exhibit high corrosion resistance, potentially addressing a significant challenge in conventional steel-reinforced structures.

The importance of corrosion protection for steel elements, particularly in façade systems, is emphasised in [18]. This review explores various methods and technologies for protecting steel elements from corrosion in building envelopes.

Steel–polymer sandwich composites represent another area of interest in advanced construction materials. The work in [19] examines the effects of interfacial adhesion on the tensile strength of these composites. By systematically varying the interfacial adhesion, the study evaluates the impact of this approach on the mechanical properties, providing insights into optimising these composite structures. The formability of steel–polymer sandwich composites is explored in [20], which introduces a new forming-limit diagram that considers both delamination and fracture. This research investigates the effect of interfacial adhesion on the formability of sandwich composites, contributing to a better understanding of their manufacturing processes and structural integrity. The work in [21] addresses the damping performance of lightweight steel/polymer/steel sandwich composites, focusing on the interface between heterogeneous materials. This study highlights the importance of considering interfacial properties in assessing the dynamic behaviour of these composite structures.

**Figure 2:**
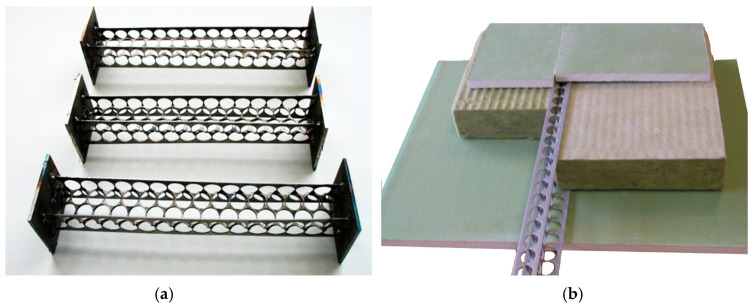
Punched-steel profiles made from the waste materials: (**a**)—Laboratory-created specimens; (**b**)—Structural members of a wall framework [19].

Punched-steel waste can be utilised to produce various profiles subjected to compression and bending forces. These profiles may be employed as structural components for walls and panels (Figure 2). Punched-steel tape can be used for fasteners, providing mechanical connections in composite timber–concrete structural members [22].

The issue of stress concentration coefficients should be considered separately in each case where punched-steel waste is used as a load-carrying component of structural members. Correct determination of the stress concentration coefficient influences structural-member dimensioning and total structural material consumption. Correspondingly, the structural efficiency of load-carrying members based on punched-metal materials (PMM) depends on total structural material consumption and the stress concentration coefficient. Therefore, the current investigation aims to increase the structural efficiency of PMM, which is composed of waste materials obtained during the stamping process in mechanical engineering. The possibility of decreasing the stress concentration coefficient should be investigated. The results should be verified through laboratory experiments and finite element (FE) models.

## 2. Materials and Methods

### 2.1. Laboratory Specimens for Determining the Coefficient of Stress Concentration

**Figure 3:**
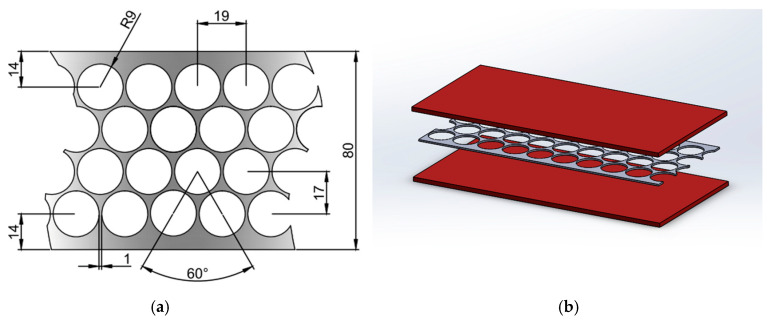
The geometric parameters of the grade LPM-4 punched-steel tape (**a**) and total view of the composite load-carrying structural element (**b**) [18].

A composite load-carrying element based on PMM waste is considered as the object of investigation in determining the stress concentration coefficient. Due to design load actions, the composite load-carrying element was developed to withstand tension forces acting in different structures. For example, it can be used as a tie-bar in timber and composite arches and as a bracing member. The composite load-bearing element is based on a punched-steel tape of grade LPM-4, produced by JSC “Ditton Driving Chain Factory” (Daugavpils, Latvia). The punched-steel tape is embedded in a polymer matrix. The geometric parameters of the tape and a total view of the composite load-carrying structural element are shown in Figure 3.

**Figure 4:**
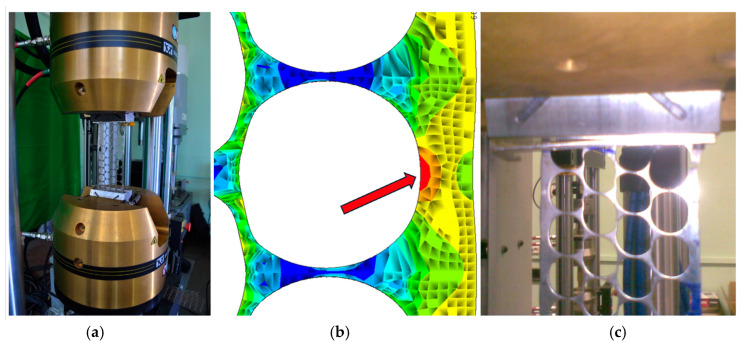
Determination of mechanical properties of LPM-4 grade punched-steel strip: (**a**)—Using the INSTRON 8802 tester; (**b**)—Zone of maximum normal stress concentration; (**c**)—Failure mode and zone of specimen collapse.

The mechanical properties of the punched-steel tape of grade LPM-4 were determined by laboratory experiments. Specimens with dimensions 300 × 80 × 1.5 mm were loaded until collapse using the testing device INSTRON 8802 (Figure 4a).

**Figure 5:**
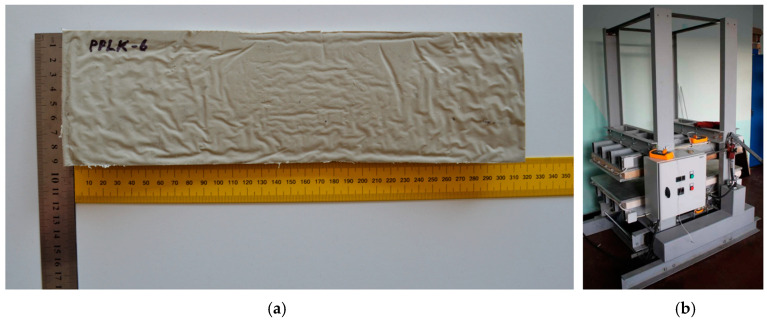
The laboratory specimen PPLK-6 (**a**) and the hot press (**b**) were used for the specimen preparation.

The zone of maximum normal stress concentration and the failure mode of the punched-steel tape specimen are shown in Figure 4b,c. The normal stress distribution in the punched-steel tape specimen was determined by the FE model described in sub-chapter 4.1. The maximum load-carrying capacity of the specimens and their strain in tension were 5.54 kN and 3.93%, respectively. The average values of the maximum tensile stress and modulus of elasticity were 220.65 MPa and 130.05 GPa, respectively. Technical thermoplastic polypropylene Polystone^®^ P grey homopolymer, produced by the German company Rochling, was considered as the matrix material. Its density, modulus of elasticity, and tensile strength are 0.91 g/cm^3^, 1.3 GPa, and 32 MPa, respectively. The thermal conductivity coefficient of the polypropilene, used for the considered composite specimen (PPLK), remained within anticipated limits, ranging from 0.17 to 0.22 W/m°K. The linear expansion coefficient remained within anticipated limits, ranging from 120 to 140 × 10^6^ K^−1^. The laboratory specimens were prepared using a hot press (Figure 5). The press is designed for products with maximum dimensions of 1200 × 600 × 600 mm. The maximum permissible temperature of the slabs for the contact surface is +350 °C. The dimensions of the prepared laboratory specimens were 310 × 90 × 6.30 mm.

Polypropylene samples were prepared with dimensions of 310 × 90 × 3 mm. The sheet size was chosen in such a way as to exceed the dimensions of the punched-steel tape LPM-4 samples, ensuring that the LPM-4 samples were fused with the polymer around the entire perimeter. The polypropylene sheet was 5 mm wider than the punched-steel tape LPM-4 sheet on each of the four sides. Polypropylene and punched-steel tape LPM-4 samples were assembled before compression to form a multi-layer sandwich according to the principle “polypropylene—punched-steel tape—polypropylene”. The considered ratio of polypropylene to steel corresponds to the minimum possible amount of the polypropylene, and will be considered as the optimum from the point of view of a specific strength of the considered composite specimens (PPLK). The sandwich was wrapped in heat-resistant food paper to limit polypropylene leakage and protect the hot-press plates. As the melting point of polypropylene ranges from 162 to 167 °C, the press was set to heat up to 210 °C, ensuring that the press plates’ surfaces would reach 180 °C. This is sufficient to melt the polypropylene. A test pressing was carried out to select the correct melt-pressing mode, during which the required plate pressure and specimen ageing time were determined. It was thus determined that for the compression of the specimens and the interlocking of the polypropylene sheets on all sides, the following regime should be used:

- Compress the sample to a load of 9.5 kN (5 bar) and hold until the pressure begins to drop due to melting of the polypropylene;
- Increase the load to 9.5 kN and expect the pressure to decrease again;
- Increase the load to 19 kN (10 bar) on the third pass, and hold the sample until the total compression time reaches 2 min;
- Unload and remove the sample from the press;
- Ensure that the press plates are interconnected before a new specimen is pressed, and the temperature returned to the predetermined level.

Six composite laboratory specimens (Figure 5a) were prepared. The composite specimens were designated PPLK-1 to PPLK-6. Five composite specimens (PPLK-1, PPLK-3–PPLK-6) were used in the experiment to investigate the coefficient of stress concentration. The sixth one (PPLK-2) was used to test the hot-press parameters described above during specimen preparation.

### 2.2. Laboratory Experiment

**Figure 6:**
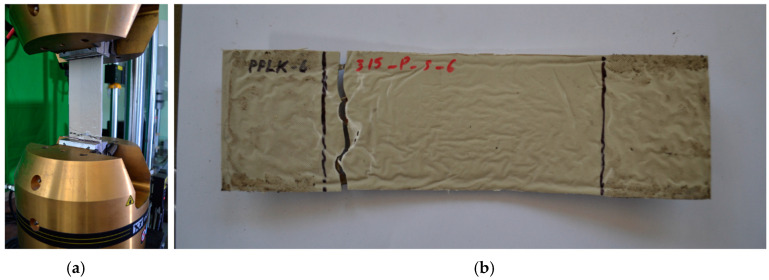
Testing the laboratory composite specimen: (**a**)—Testing device INSTRON 8802; (**b**)—Laboratory composite specimen PPLK-6 after it had collapsed.

The laboratory experiment was conducted using the testing device INSTRON 8802 (Figure 6a). The composite specimens’ fixation length in the testing device was 60 mm on each side. The composite specimens’ working zone length was 180 mm (Figure 6a). The loading velocity of the composite specimens was 5 mm/min.

All five composite specimens (PPLK-1, PPLK-3—PPLK-6) were loaded with an axially applied tension breaking force until failure. The shape of specimen PPLK-6 after its failure is shown in Figure 6b. The same mode of failure was observed for all five specimens. Zones of specimen failure were observed quite close to the fixation zones in the testing device but always within the working zone of the specimens. It was noted that the polymer matrix and the punched-steel tape component broke simultaneously.

### 2.3. Analysis of Laboratory Samples

The analysis of the laboratory specimens was joined with the determination of their load-carrying capacity and modulus of elasticity.

The load-carrying capacity of the laboratory specimens PPLK was determined based on the principle of proportional summation. This means that the load-carrying capacity of the laboratory specimens was determined as the sum of the polypropylene and steel load-carrying capacities, as shown in Equation (1):

(1) F_(t,max,pplk)=2F_(t,max,pp)+F_(t,max,pl)

where *F_t, max,pplk_*—load-carrying capacity of laboratory specimens, kN; *F_t, max, pp_*—load-carrying capacity of polypropylene component, kN; *F_t, max, pl_*—load-carrying capacity of punched-steel tape component, kN.

The maximum load-carrying capacity of the laboratory specimen was equal to 19.19 kN. The maximum load-carrying capacities of the polypropylene and punched-steel tape components were equal to 6.83 kN and 5.53 kN, respectively.

As the composite (PPLK) is composed of transversely isotropic materials, the final product was assumed to be transversely isotropic. In the case of a transversely isotropic material, the elastic modulus was determined using Equations (2) and (3).

(2) E_1=ψE_pl+(1−ψ) E_pp

(3) E_pplk= (E_pl E_pp E_1)/(E_1 [ψE_pp+(1−ψ)E_pl ]−ψ(1−ψ) 〖(∂_pp E_pl−∂_pl E_pp)〗^2)

where *E*_1_—approximate value of the modulus of elasticity of the laboratory specimen, taking into account the percentages of the different materials (volume fractions), GPa; *E_pplk_*—modulus of elasticity of the laboratory specimen, taking into account the approximate modulus of elasticity and Poisson’s ratios of each of the constituent materials of the composite, GPa; *E_pl_*—modulus of elasticity of punched-steel tape, GPa; *E_pp_*—modulus of elasticity of polypropylene tape, GPa; Ψ—volume fraction of the punched-steel tape in the cross-section of the laboratory specimen; ϑ_pp_—Poisson’s ratio for polypropylene; ϑ_pl_—Poisson’s ratio for punched-steel tape.

The value of the volume fraction of punched-steel tape in the cross-section of the laboratory specimen was equal to 0.046. The Poisson’s ratios for polypropylene and punched-steel tape were taken to be 0.41 and 0.60, respectively. The approximate value of the modulus of elasticity of the laboratory specimen was equal to 7.50 GPa. The modulus of elasticity of the laboratory specimen, taking into account the approximate modulus of elasticity and the Poisson’s ratios of each constituent material of the composite, was equal to 1.57 GPa. The coefficient of stress concentration was determined as the relation of the maximum normal stresses to the nominal value of the normal stresses, equal to the relation of the axial force acting on the member to the effective area of the member’s cross-section, determined while taking the perforation into account. The maximum stresses in the punched-steel plate components should not exceed 290 MPa, so the ultimate tensile strength of the steel was used for this determination.

## 3. Results and Discussion

The experiment found that in the polypropylene layer before the PPLK collapse, microcracks are formed which replicate the contour lines of the perforation slits in the tape. However, the material does not collapse and continues to deform. This results in a simultaneous loss of structural capacity; in this experiment, the LPM-4 and polypropylene components lost their load-carrying capacity at the same moment the LPM-4 tapes collapsed. It should be noted that this failure mode was typical and was observed for all PPLK laboratory specimens. For example, Figure 6b shows the failure mode for PPLK-6.

In some places, the adhesion between the metallic and polymeric components was not sufficiently strong, resulting in delamination between the components. The delamination occurred due to poor adhesion, which was also anticipated. The interlocking of the structure was ensured by the penetration of the polypropylene into the perforation slots and mutual adhesion. Adding chemically active agents could probably solve the problem of adhesion improvement. On the other hand, the perforation slots that were filled with a polymer remained tightly bound to each other.

**Figure 7:**
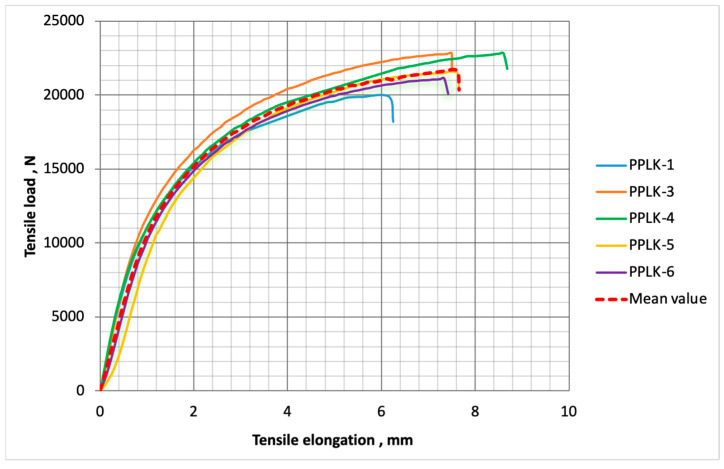
The dependences of the tensile elongation of the PPLK specimens on the axial tensile load.

The load-carrying capacities of the specimens changed, within anticipated limits, from 19.93 to 22.82 kN. The tensile elongation of the specimens changed, within anticipated limits, from 3.43 to 4.78%. The mean values of the load-carrying capacity and strain were equal to 21.64 kN and 4.14%, respectively. The moduli of elasticity of the PPLK specimens changed, within anticipated limits, from 2.79 to 3.74 GPa. The mean value of the modulus of elasticity was equal to 3.38 GPa. The dependences of the tensile elongation of the PPLK specimens on the axial tensile load are shown in Figure 7.

**Figure 8:**
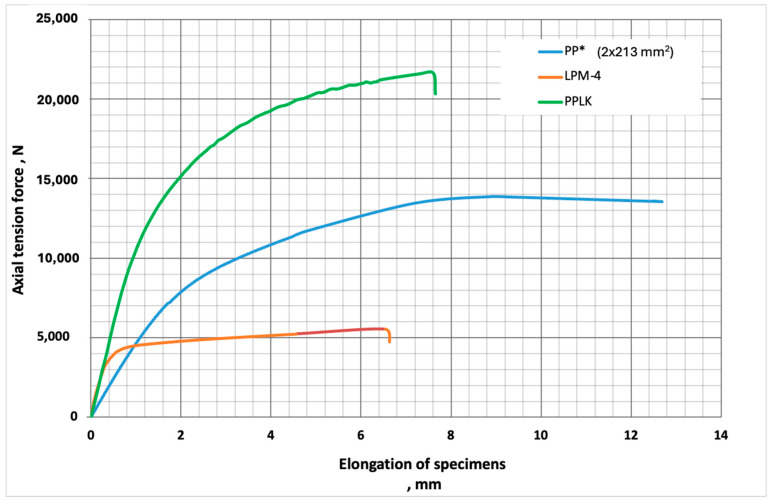
The dependence of axial tension force on the elongation of the composite PPLK specimens.

The maximum difference between the results obtained by the analytical approach and the experiment was observed for the modulus of elasticity of the PPLK specimens; it was equal to 53.55%. The analytical approach was based on the method of the summing of the moduli of elasticity and the tensile strengths of steel and polypropylene in proportion to its volume fractions—the so-called method of proportion summing. This approach is generally used for the analytical prediction of the mechanical properties of hybrid composite structural members working in tension, and a good correspondence was observed in the results obtained by the experiments [23]. The increase of the experimental value of the modulus of elasticity of the composite specimen, in comparison with the value obtained by the analytical calculations, was due to the redistribution of stresses between the punched-steel tape and polypropylene, which also increased the load-carrying capacity of the composite specimen. Comparison of the analytical calculations and the experimental results indicates that the experimentally determined values are higher than those calculated analytically. This suggests that the polypropylene and punched-steel tape in the composite specimens mutually improve each other’s properties. The dependence of the axial tension force on the elongation of the composite PPLK specimens is given in Figure 8.

The elastic modulus of the PPLK composite specimens decreased relative to the modulus of elasticity of the punched-steel tape LPM-4 component. Hence, the deformation of the composite specimen increased compared to the deformations of the punched-steel tape.

The load-carrying capacity of the PPLK composite specimen (21.64 kN) is higher than the sum of the load-carrying capacities of its constituent components (19.18 kN). This means that the performance of the individual materials in the PPLK composite specimen increased the total load-carrying capacity of the composite specimen by 2.46 kN, or 11.37%. The increased load-capacity is due to the redistribution of stresses in the polypropylene and its local failure, not only along the perforation contour but also inside it. This means that the behaviour of the PPLK composite specimen also was related to the polypropylene material filling the perforation slots. The filling of the perforation slots also influenced the stress redistribution in the punched-steel band and decreased the coefficient of stress concentration. The load-carrying capacity of the PPLK composite specimen depends on the mechanical properties of the matrix and, first of all, on its modulus of elasticity. Using matrices with an increased value for the modulus of elasticity can enable the reduction of stresses in the punched-steel tape. It allows an increase in the structural efficiency of punched-metal-materials-based composites. This question is considered in Section 4 of the current study.

## 4. Finite Element Analysis of the Structural Efficiency of Composites Based on Punched-Metal Materials (PMM)

### 4.1. Description of FE Model

**Figure 9:**
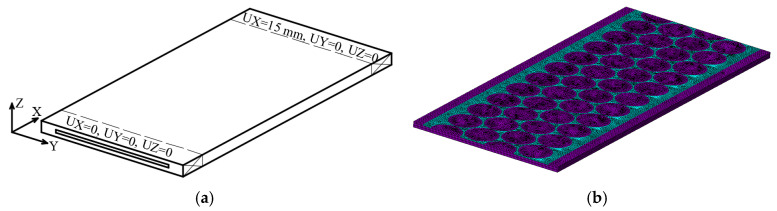
FE model of the PPLK composite specimen: (**a**)—Scheme with applied boundary conditions; (**b**)—FE model mesh (symmetric half of specimen’s thickness is shown).

The stress distribution in the PPLK composite specimen was analysed numerically using the finite element method (FEM) and realised by the software ANSYS 2023R2. The FE model was built in ANSYS Mechanical APDL software using SOLID185 finite elements for both steel and polypropylene. For modelling of steel, part 84192 FEs were used; the polypropylene part was represented by 162838 FEs. Interaction between parts made of different materials was modelled under standard contact conditions allowing separation and sliding with friction. TARGE170 and CONTA174 finite elements were utilized for definition of the symmetric surface-to-surface contact pair. The dimensions of the FE model were 208 × 89 × 6.4 mm. Boundary conditions were applied to the first 14 mm from both sides of the specimen in longitudinal direction. From one side of the specimen, all nodal displacements were restricted, and from the second side, both transversal displacements were restricted and a displacement equal to 15 mm was defined in a longitudinal direction. Thus, the finite element model was loaded by displacement. This loading condition corresponds to the loading of the specimen in the experimental equipment. At the same time, the loading of the FE model by displacements allowed researchers to ensure a more reliable convergence of nonlinear static analyses, taking into account both geometrical and material nonlinearities. The force in the numerical specimen was calculated as the sum of the reaction forces. The FE model of specimen is presented in Figure 9.

**Figure 10:**
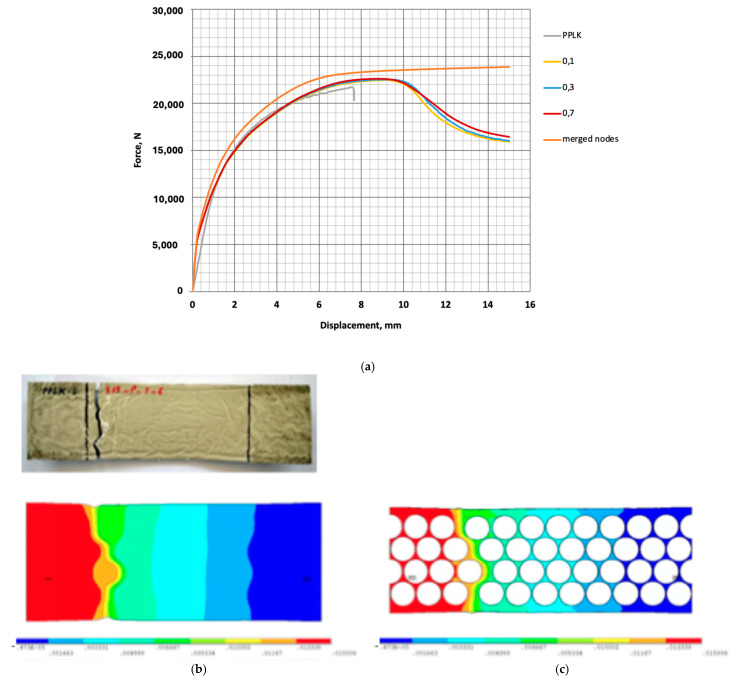
PPLK composite specimen behaviour analysis with the FE model: (**a**)—Axial tension force as a function of the elongation in tension; (**b**)—The experimental and numerical deformed shape of the specimen after failure; (**c**)—The numerical deformed shape of steel after failure.

A parametric study on the friction coefficient between steel and polypropylene was performed. The results obtained using friction coefficients of 0.1, 0.3, and 0.7 were compared. No significant effect of the value of the friction coefficient on the behaviour of the composite sample model was found. The results of the PPLK behaviour analysis for the suggested FE model are shown in Figure 10.

Numerically, the load-bearing capacity was the same for all friction coefficients. A model with merged steel and polypropylene nodes on the contact surfaces was calculated for comparison. As expected, it showed higher stiffness and strength of the composite sample. The finite element model was loaded by displacement, as it is in the experimental design. The force in the numerical specimen was calculated as the sum of the reaction forces. The FE model developed in this manner can be used to predict the behaviour of the PPLK composite specimens.

**Figure 11:**
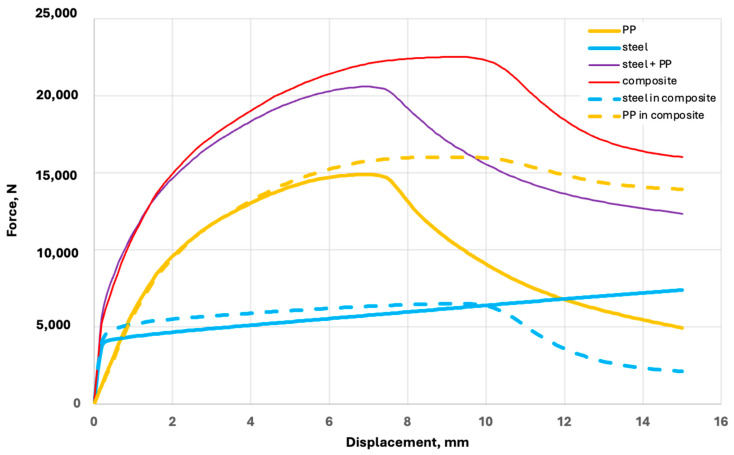
Comparison of results obtained by the FE models for steel, polypropylene, a sum of steel and polypropylene, and PPLK composite.

The dependence of the axial tension force and elongation (displacements) obtained by the FE models for the components of the PPLK composite specimen (steel and polypropylene), for the steel and polypropylene sum (of steel and polypropylene), and the PPLK composite specimen are shown in Figure 11.

In Figure 11, “PP” is a result obtained for the separate polypropylene component, “Steel” is a result obtained for the separate steel component, and “steel + PP” is a result obtained by summing the results obtained for polypropylene and steel. Three other results, “composite”, “steel in composite”, and “polypropylene in composite”, were obtained by the FE model analysis of the PPLK composite. The results obtained by the FE model confirm that the PPLK composite can withstand a higher axial tension force than can the sum of the steel and polypropylene. This can be attributed to the decrease in the coefficient of stress concentration.

### 4.2. Decrease the Coefficient of Stress Concentration in the Steel Component of the PPLK Composite

**Figure 12:**
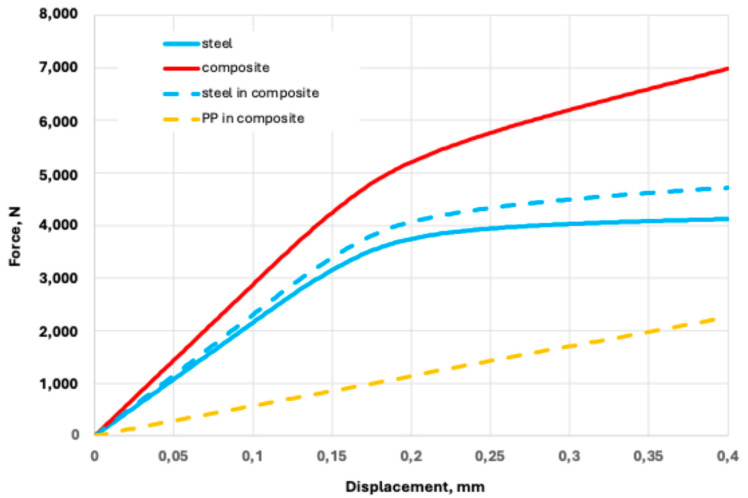
Axial force as a function on the displacements applied to the composite specimen: the punched-steel tape, in the composite and separately, and polypropylene.

Additional analyses of the composite specimen, the punched-steel tape in the composite separately, and the polypropylene by the FE model were carried out to analyse the behaviour of the steel in the context of elastic work. The tension force was applied as the corresponding value of the displacements. The PPLK composite specimen and its components were subjected to absolute deformation (displacements) up to 0.4 mm, with a step of 0.002 mm. Initially, the FE models of the PPLK composite specimen and the punched-steel tape were subjected to an absolute elongation of 15 mm applied with a step of 0.1 mm. Results for the axial force N as a function of the displacements (mm) applied to the composite specimen, namely, the punched-steel tape, in the composite and separately, and the polypropylene, are shown in Figure 12.

**Figure 13:**
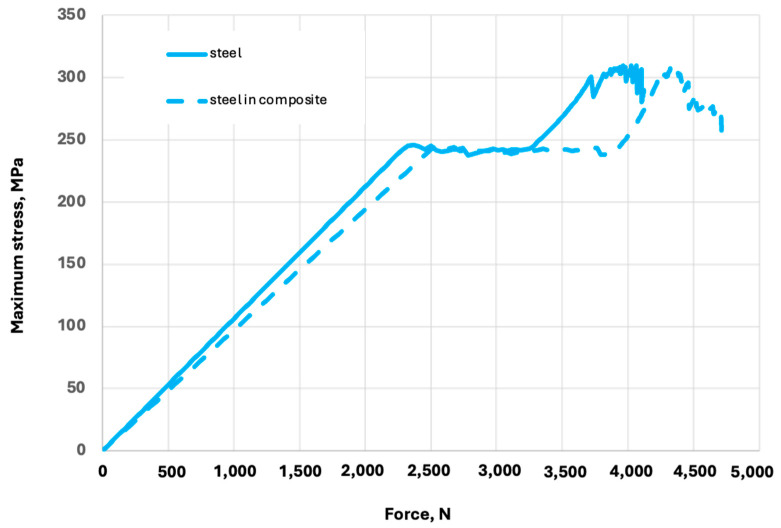
The maximum stresses in the punched-steel tape as a function of the axial force, separately and in the PPLK composite specimen.

At the deformation of 0.4 mm, the stresses in the steel component (punched-steel tape) of the PPLK composite specimen reach the yield point. Of primary interest is comparing the behaviour of the punched-steel tape, separately and in the PPLK composite specimen. The maximum stresses in the punched-steel tape as a function of the axial force are shown in Figure 13.

**Figure 14:**
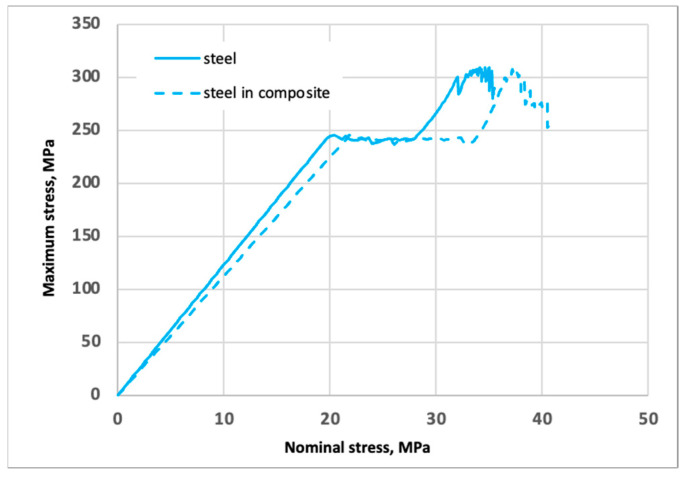
The maximum stresses (in Mpa) are a function of the nominal stresses (in Mpa) in the separate punched-steel tape and the steel component of the PPLK composite specimen.

The point of yielding in the separate punched-steel tape and the steel component of the PPLK composite specimen occurs at axial forces of 2312.5 N and 2562.5 N, respectively. So, the influence of the polypropylene matrix increases the load-carrying capacity of the punched-steel tape component and the whole PPLK composite specimen. Then, taking into account the dimensions of the punched-steel tape cross-section, 77.5 × 1.5 mm without perforations, the maximum stresses (MPa) as a function of the nominal stresses (Mpa) in the separate punched-steel tape and the steel component of the PPLK composite specimen can be obtained (Figure 14).

**Figure 15:**
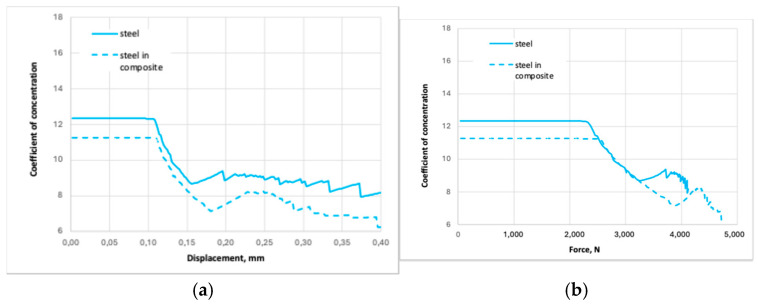
The coefficient of stress concentration as a function of the displacements (**a**) and the axial tension force (**b**).

Suppose we consider the coefficient of stress concentration as the ratio of the maximum stress to the nominal stress. In that case, we can derive its value as a function of the displacement (we can go from displacements to deformations). The coefficient of stress concentration as a function of the displacements is shown in Figure 15a. The coefficient of stress concentration as a function of the axial tension force is shown in Figure 15b.

**Figure 16:**
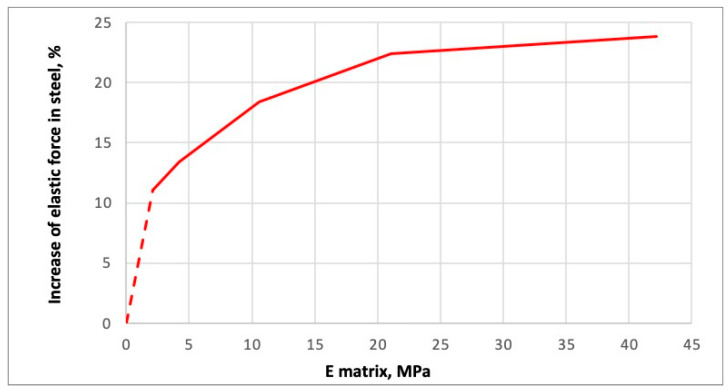
The increase in the “elastic” force (axial force) in the punched-steel tape is a function of the matrix modulus of elasticity.

Additional analyses were performed, with the matrix elastic modulus increased up to 42 MPa to evaluate the stress concentration coefficient. The dependence shown in Figure 16 displays the increase in the “elastic” force (axial force) in the punched-steel tape as a function of the matrix modulus of elasticity. The maximum elastic force, in this case, is determined when the increase in force is no longer proportional to the increase in applied displacement (the increase in force with a uniform increase in applied displacement decreases to the initial rise in force by more than 1%). Thus, this graph shows the increase in axial force at which the behaviour of the punched-steel tape becomes plastic. The appearance of individual plastic zones in places of stress concentration at the edges of the holes is not considered here.

**Figure 17:**
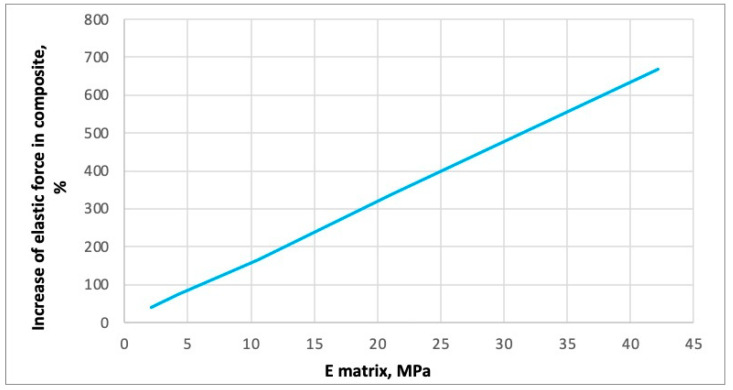
The elastic force increases in the PPLK composite specimen at the moment of steel-to-plastic transition changes as a function of the modulus of elasticity of the matrix.

It should be noted that the forces at which the first plastic zones appear are less than the analysed ones by only 2–3% (i.e., local zones fuse, and the whole section becomes plastic with a slight increase in the force). The dependence of the magnitude of these forces on the matrix elastic modulus is the same. Thus, this graph, shown in Figure 16, is valid for describing the change of forces in steel at which the first plastic zones appear in the places of stress concentration. So far, in the parametric study of the effect of matrix stiffness, we have not considered how the forces in the PPLK composite specimen change. It is assumed that the matrix works elastically. Then, the forces in the PPLK composite specimen at the moment the steel transitions to plastic behaviour change, as shown in Figure 17.

It was demonstrated that an increase in the modulus of elasticity of the matrix up to 42 MPa results in a 24% enhancement in the elastic force in the steel component of the PPLK composite specimen, compared to the separate punched-steel tape. The elastic force in the composite increases by a factor of six. This demonstrates that the efficiency of the punched-steel tape in composite materials is contingent upon the modulus of elasticity of the matrix, as the latter exerts an influence on the coefficient of stress concentration. The coefficient of stress concentration declines in proportion to the augmentation of the elastic force in the composite.

## 5. Conclusions

The present study demonstrates the feasibility of incorporating LPM-4-grade steel tape waste into a PPLK composite (310 × 90 × 6.30 mm) for structural applications. The composite shows improved load-carrying capacity, with an experimental average of 21.64 kN, exceeding the theoretical 19.18 kN by 11.37%. The average modulus of elasticity (3.38 GPa) represents a 2.5-fold increase over the polypropylene matrix (1.3 GPa).

FEA confirms stress mitigation in the steel component. Increasing the matrix’s elastic modulus to 42 MPa resulted in a 24% increase in the steel component’s elastic force. Five specimens (PPLK-1, PPLK-3–PPLK-6) showed simultaneous failure of steel and polymer components.

The composite’s performance is sensitive to the polymer matrix characteristics. Load-carrying capacity ranged from 19.93 to 22.82 kN, with some delamination observed. A 53.55% discrepancy between the analytical and experimental modulus of elasticity values was noted.

Future research should focus on improving interfacial adhesion, optimizing manufacturing processes, and exploring different geometries to enhance performance and consistency.

## Data Availability

The data that support the findings of this study are available from the first author [M.L.] upon request.
